# Epilepsy Videos on YouTube: A Cross-Sectional Observational Study

**DOI:** 10.7759/cureus.43916

**Published:** 2023-08-22

**Authors:** Netra Bhoot, Aasvi V Gohil, Kapil Usgaokar, Keyur Ranpariya, Rakshit Yadav, Ankita Nanda

**Affiliations:** 1 Internal Medicine, Jawaharlal Nehru Medical College, Belgavi, IND; 2 Pediatrics, Gujarat Medical Education and Research Society (GMERS) Medical College and Hospital, Vadodara, IND; 3 Hospital Medicine, Sussex Partnership National Health Service (NHS) Foundation Trust, Chichester, GBR; 4 Pediatrics and Neonatology, UNM Children's Hospital, Gujarat, IND; 5 Internal Medicine, Ram Kishan Yadav (RKY) Memorial Hospital, Jaipur, IND; 6 Medicine and Surgery, Rangaraya Medical College, Kolkata, IND

**Keywords:** discern tool, global quality score, epilepsy cure, epilepsy cause, epilepsy prevention, epilepsy treatment, seizures, youtube, epilepsy

## Abstract

Introduction: Epilepsy is defined as a disorder of the brain characterized by an enduring predisposition to epileptic seizures. Being the most common neurological condition in the world, information regarding epilepsy is gathered by people from different available sources.

Objectives: The objective of this study was to evaluate the reliability and quality of YouTube videos about epilepsy as a source of information for the general population and also for patients suffering from this illness and for their families.

Methodology: A cross-sectional observational study was conducted, utilizing a questionnaire prepared on Google Forms (Google LLC, Mountain View, California, United States) with predetermined criteria. Each of the six authors independently searched and evaluated 10 YouTube videos using specific keywords. The assessment included determining the global quality score and utilizing the DISCERN tool. The collected data was recorded in Microsoft Excel and subsequently analyzed.

Results: A total of 60 videos were analyzed, revealing that 76.27% of them provided information on the available treatment options for epilepsy, 71.19% explained the cause and etiology of the condition, and only 3.39% of the videos mentioned support groups.

Conclusions: Our study highlights the importance of assessment of medical information on social media platforms in order to ensure availability of correct information with high quality and reliability for epilepsy patients. This will help in understanding their medical health issues and decision making.

## Introduction

Epilepsy is a neurological disorder characterized by the occurrence of recurrent unprovoked seizures. Epilepsy is diagnosed in the case of a minimum of two seizures without a known trigger, with a 24-hour interval between them. This condition is noncontagious and encompasses various forms with distinct features and causes. With 50 million known cases worldwide, epilepsy ranks among the most prevalent neurological disorders. The ratio of individuals in the general population with intractable epilepsy, or those experiencing ongoing seizures or epilepsy requiring anti-seizure medication, ranges from four to 10 per 1000 people. As a result, epilepsy has a significant global impact [1].

With the substantial global burden of epilepsy, individuals often seek information from various sources to gain a better understanding of the disease. One commonly utilized platform for information retrieval is YouTube, which boasts approximately 122 million daily users and 2.1 billion monthly active users worldwide [2]. Consequently, it is crucial to evaluate the data provided on this platform, particularly for a condition that is often misunderstood and can profoundly impact the quality of life. Many doctors, either working independently or affiliated with hospitals, share their expert advice on this social platform. Such advice may be biased, aiming to attract attention to their respective medical institutions, or it may be unbiased, solely intended to disseminate information to the general public without any hidden motives.

The present study aimed to assess the quality and reliability of information available on YouTube concerning epilepsy causes, prevention, and treatment options (including both pharmacological and surgical approaches), among others. To eliminate any potential influence from prior searches, the researchers deleted cookies, cache, and search history from Google and YouTube before initiating the video search process, ensuring that the new samples used in this study were not impacted by previous search preferences.

Aims and objectives

In today's era where YouTube is widely used as a reference for medical information, this study aimed to analyze the quality and reliability of the data available on YouTube about epilepsy as a source of information for the general population and especially its usefulness for patients suffering from this illness and for their families. In addition to the content, this study also aimed to provide insight into viewer engagement and a variety of sources of epilepsy videos.

## Materials and methods

This study was a cross-sectional observational study conducted on a single day, specifically on the 23rd of March 2023. Institutional approval was not required for our study as it did not involve any human subjects.

A questionnaire was created on Google Forms (Google LLC, Mountain View, California, United States) with predetermined criteria, focusing on three broad characteristics. First, we examined the basic characteristics of the videos, including the source of the uploaded videos and the time frame of the posts. Second, we analyzed the type of information presented in the videos, looking for descriptions of symptoms, causes, investigations, prevention, treatment, mortality, rehabilitation, and personal experiences shared by patients and their family members. We also considered if the post contained promotional content by pharmaceutical companies or doctors. Lastly, we assessed the quality and reliability of the videos using the Global Quality Score (GQS) and DISCERN tool, respectively [3].

Each of the six authors conducted searches and evaluated 10 YouTube videos using specific keywords, such as "epilepsy," "seizures," "epilepsy treatment," "epilepsy prevention," "epilepsy cause," and "epilepsy cure." The inclusion criteria required relevance to the topic, language suitability, and a video length ranging from one to 20 minutes. Videos that did not meet the inclusion criteria were excluded from the study, and any duplicate entries were removed.

The collected information was entered in Microsoft Excel (Microsoft Corporation, United States), and statistical analysis was done using IBM SPSS Statistics for Windows, version 21 (Released 2012; IBM Corp., Armonk, New York, United States).

## Results

A total of 60 videos were initially assessed. After applying the inclusion and exclusion criteria and removing duplicate videos, a total of 59 videos were considered and analyzed for various variables. The popularity of the videos was determined by evaluating different variables, including the number of views, number of likes, number of dislikes, and number of comments. Table 1 presents the results for the popularity of the analyzed videos. 

**Table 1:** Popularity of the analyzed videos

Variables	N
Total no. of views	12,975,177
Total no. of likes	132,722
Total no. of dislikes	4,652
Total no. of comments	7,881

Moreover, two additional characteristics were assessed: the time elapsed since the videos were uploaded and the ownership of the channel that posted the videos, such as doctors and healthcare organizations. Table 2 presents the results for these characteristics.

**Table 2:** Characteristics of the videos

Time since the video was uploaded	Total no. of videos N (%)
More than a week to one month (7-30 days old)	01 (1.7%)
More than a month to six months (31-180 days old)	04 (6.8%)
More than six months to last one year (180-365 days)	07 (11.9%)
More than one year (>365 days)	47 (79.7%)
Video uploaded by	Total number N (%)
Doctors	07 (11.9%)
Hospitals	18 (30.5%)
Healthcare organizations	04 (6.8%)
News channels	03 (5.1%)
Others	27 (45.8%)

Furthermore, an analysis was conducted on the information being conveyed by the videos. Table 3 illustrates that the YouTube videos discussed about etiology, treatment, symptoms, and prevention, among others. 

**Table 3:** Information about epilepsy in the videos

Information	N (%)
Symptoms	32 (54.24%)
Aetiology/cause	42 (71.9%)
Investigations/tests	21 (35.59%)
Treatment	45 (76.27%)
Prevention/vaccines	26 (44.07%)
Mortality	05 (08.47%)
Rehabilitation	05 (08.47%)
Support groups	02 (03.39%)
Patient/people sharing their own experience	08 (13.56%)
Parents sharing their experience with their family	03 (05.08%)
Promotional content by pharmaceutical companies or by doctors	06 (10.17%)

A comparison was performed based on the Global Quality Score (GQS), reliability, and video power index (VPI) for the evaluated videos. The results of this comparison are presented in Table 4.

**Table 4:** Comparison of the GQSs, reliability scores, and VPIs based on the type of uploader (n=60) Median values are presented as Median (IQ1, IQ3), where IQ1 represents the first quartile and IQ3 represents the third quartile. The p-value and the test used are also provided. The Kruskal-Wallis test was used for the statistical analysis. GQS: Global Quality Score, VPI: video power index. *x* signifies categories that are either too skewed or have too little number of values, hence having no Q3.

	Doctors (n=11) Median (Q1, Q3)	Hospitals (n=5) Median (Q1, Q3)	Healthcare organizations (n=7) Median (Q1, Q3)	News agency (n=28) Median (Q1, Q3)	Others (n=9) Median (Q1, Q3)	P-value and the test used
VPI	8.7 (0.68, 72.20)	10.0266 (4.8256, 122.0773)	36.8021 (17.7921, 262.5296)	0.509 (0.2717, *x*)	41.1477 (9.2322, 161.9802)	0.098
GQS	3 (2, 4)	3 (2, 4)	3.5 (2.25, 4)	4 (4, *x*)	3 (2, 4)	0.164
Reliability score	3 (2, 4)	3 (2, 3)	4 (2.5, 4)	3 (3, 3)	3 (2, 3)	0.486

## Discussion

Epilepsy is a chronic neurological condition characterized by recurrent unprovoked seizures caused by abnormal electrical signals in the brain. The imbalance of excitatory and inhibitory neurotransmitters can contribute to these seizures, and the expression of receptors and ion channels triggered by seizure-inducing neurotransmitters may vary [4].

The prevalence of epilepsy is relatively high, with approximately 3.4 million individuals actively affected, including three million adults and 470,000 children. Population-based studies indicate that at least 50% of children who develop epilepsy during childhood experience remission [5]. The stigma associated with epilepsy has been linked to reduced quality of life for affected individuals [6]. Thus, raising awareness about epilepsy is crucial, and YouTube serves as a popular medium for disseminating information [7].

However, studies have indicated that health-related content on YouTube tends to have average to below-average quality, making it an unreliable source of health information [8]. Despite this, many people rely on YouTube videos for information on epilepsy. Therefore, it is essential to evaluate the quality, reliability, and reach of epilepsy-related videos on YouTube.

This study aims to assess the accuracy, dependability, and comprehensiveness of epilepsy-related materials available on YouTube. We analyzed 60 videos on YouTube that provided information about epilepsy and seizures, examining their impact on the viewing audience. The 60 videos collectively received 12,975,177 views, 132,722 likes, 4,652 dislikes, and 7,881 comments. Among the analyzed videos, 76.27% highlighted treatment options for epilepsy, and 71.19% explained the causes and etiology of epilepsy and seizures.

By contrast, only 3.39% of the videos mentioned support groups. Most videos were over a year old but still had considerable reach within the YouTube audience. Educational channels accounted for 45.8% of the videos, followed by hospitals (30.5%), doctors (11.9%), healthcare organizations (6.8%), and news agencies (5.1%).

In our study, 54.24% of the videos covered symptoms, while another study assessing 100 videos reported only 28% coverage of symptoms [9]. Furthermore, another study found that 46% of videos were uploaded by doctors and 33% by healthcare channels, contrasting with our study's findings of 11.9% by doctors and 45.8% by educational media [10]. Our study yielded a mean GQS of 3.3 and a mean reliability/DISCERN score of 3.2. Another study reported a mean GQS of 3.21 ± 1.05 and a mean DISCERN score of 3.71 ± 1.17 [10]. In addition, our study introduced the calculation of the VPI for each uploader, which was not addressed in previous similar studies. A study focusing on miniaturized percutaneous nephrolithotomy (mPCNL) and YouTube videos reported mean VPI, GQS, reliability, and mPCNL scores of 4.61, 2.86, 2.61, and 9.58, respectively [11]. In a separate study on YouTube videos about ovarian cysts, reliability and general quality scores were found to be 2.81 ± 1.3 and 2.88 ± 1.4, respectively [12].

Despite significant advancements in epilepsy research and treatment, such as gene discovery and diagnosis [13,14], epilepsy remains a significant concern for the society, significantly impacting the quality of life for patients and their families [15,16]. Since social media is used to access healthcare-related information not only about epilepsy, similar to several other diseases [11,12], government agencies and healthcare organizations should join hands to form rules to have strict actions against those circulating unverified and unreliable information on social media, such as YouTube. 

Limitations

The limitations of our study include the analysis of only 60 posts over a specific period, while new posts are regularly uploaded, potentially affecting the number of views, likes, dislikes, and comments for each video. There is also the possibility of different search results at different times and interobserver variation, as a single video's GQS may vary depending on the evaluator. However, this might only slightly affect the outcome.

## Conclusions

Our study highlights the importance of assessment of medical information on social media platforms. This will ensure that consumers have access to accurate and trustworthy information, which is essential in their decision-making regarding their health. Future studies in this field should concentrate on formulating plans to encourage the distribution of correct medical information and thwart the spread of false information on social media platforms. Nonetheless, more research is required to ascertain the long-term effects of false information on public health and to help shape public health policies that are intended to lessen these effects.
